# Patient’s Perspective on Factors Affecting Health-Seeking Behavior in Al-Ahsa, Saudi Arabia

**DOI:** 10.7759/cureus.30078

**Published:** 2022-10-08

**Authors:** Abdullah Almaqhawi, Shahad Alsayil, Mohammed Al Qadhib, Ahmed Alkhawfi, Abdullah Alkhalaf, Zahra Al Khowildi

**Affiliations:** 1 Family and Community Medicine, College of Medicine, King Faisal University, Al-Ahsa, SAU; 2 Medicine, King Faisal University, Al-Ahsa, SAU; 3 General Physician, King Faisal University, Al-Ahsa, SAU

**Keywords:** factors, saudi arabia, primary health care, behavior challenges, health seeking

## Abstract

Background

There are different determinants of health-seeking behaviors. Studying health-seeking behaviors and their factors help governments use the existing health resources properly for a potent healthcare system. This study aimed to evaluate the factors influencing health-seeking behavior in Al-Ahsa.

Methods

The study is a questionnaire-based observational cross-sectional study of the factors influencing health-seeking behaviors in the general population of the Al-Ahsa governorate. A non-probability convenience sampling technique was used to acquire the sample. The study followed all ethical considerations and received approval from King Faisal University.

Results

A total sample of 481 participants completed the study questionnaire. 21.2% of people visited the health centers for medical checkups despite having no symptoms, while 29.1% admitted to avoiding the health centers despite knowing they needed medical attention. Additionally, the majority of patients prefer government-run health facilities (58.6%), while 41.4% prefer the commercial sector. When experiencing any health complaints, precisely 70.7 % of women go to a medical facility, compared to 61.8% of men (P=.038). In addition to that, 68.5% of participants with intermediate economic status visit healthcare centers for any symptoms compared to 50% of others with high financial status (P=.049). Specifically, 73.3% of participants diagnosed with a disease or chronic diseases visited healthcare centers for clinical symptoms compared to 64.3% of others without (P=.049).

Conclusion

This study identified that most participants with chronic diseases seek medical care for any symptom, and the participants with an intermediate economic status are more likely to go to a governmental primary healthcare center for any symptoms. The findings of this research provide insights for the government and policymakers to create effective strategies and manage the existing resources in Al-Ahsa.

## Introduction

Saudi Arabia has shown dramatic increases in chronic disease burden [1]. Saudi Arabia has the seventh rate of diabetes mellitus in the world [2,3]. The prevalence of hypertension and hypercholesterolemia has significantly increased in men and women [4], contributing to high health costs for the government. For instance, the recent Diabetes estimated cost reached up to 4.5 billion USD [5]. Saudi Arabia has set a 2030 vision that includes all the directions, goals, and commitments that Saudi Arabia seeks to achieve in various fields [6]. One of these goals is to develop the health sector. This goal aims to facilitate access to healthcare services, promote the prevention of health risks and improve the value of health services [6]. Using effective healthcare will minimize the disease burden and enhance people's general health [7] through universal health coverage. Therefore, it will decrease the economic burden on the country [5]. The healthcare-seeking behaviors reflect healthcare utilization and its patterns. The health-seeking behaviors determine the community's utilization of healthcare services and the impact of this usage on the population [8]. Therefore, the provision of health services should be based on knowledge of healthcare consumption and seeking behaviors, as well as the variables that influence these behaviors in the context of political, economic, social, and cultural beliefs, as well as on gender norms and religious practices [9]. Government officials, legislators, and health service providers can distribute and manage available resources more effectively by understanding healthcare-seeking behaviors and their causes [10]. Currently, there is limited research regarding barriers to health-seeking behavior in the eastern provision, Saudi Arabia. Therefore, this study aimed to evaluate the factors influencing health-seeking behavior in Al-Ahsa, Saudi Arabia.

## Materials and methods

Study patients 

The general population for this study included all Al-Ahsa residents between the ages of 18 and 65. The exclusion criteria were participants younger than 18 years and older than 65 years, and the presence of mental disabilities prevented the survey filling.

Sampling technique

The sample was collected using a non-probability convenience sampling technique from November 1, 2021 to December 1, 2021. The questionnaire was created and distributed using a Google form online questionnaire. The link for this survey was sent via social media (Twitter, Facebook, and WhatsApp).

Study procedure

The current study is a questionnaire-based observational cross-sectional study of factors influencing health-seeking behaviors in the general population of the Al-Ahsa governorate. The participants were asked about the behavioral challenges during their visit to the healthcare center.

Using a modified questionnaire from a previous study, the researchers created a valid and accurate questionnaire [11]. It was adapted from previous research and had a section for giving informed consent and protecting the participants' privacy. In addition, consultants' reviews and the intention to verify reliability were used in a pilot study of the first 10 responses using Cronbach's alpha test. The questionnaire contained information about predisposing factors such as age, gender, marital status, number of children, family size, nationality, and education level. The enabling elements were discussed in questions that included insurance status, self-rated economic position, monthly income, employment status, and types of workplaces. Health insurance status was one of the most important elements impacting people's health-seeking behavior.

Monthly income of participants was assessed. The answers were categorized into three groups “less than 4,000 Saudi riyals,” “between 4,000 and 14,000 Saudi riyals,” and “≥ 14,000 Saudi riyals.” insurance status is directly correlated with employment status and workplace characteristics, which affect access to and preference for health services. The following questions are regarding the need factors of the participants, including self-reported health status, history of having chronic diseases, medicine intake, and chronic diseases in the family (Yes/No). Self-reported health status was assessed through the question “How do you rate your general health?” with the options “excellent,” “very good,” “good,” “average,” and “poor.” For the analysis, the answers will be classified into three groups “good” (including excellent, very good, and good), “fair,” and “poor.”

History of having a chronic disease is assessed with the question “Have you ever been diagnosed by a doctor with one or more chronic illnesses?” with the answer options “Yes” and “No.” The information on medicine intake regularly is obtained through the question “Do you take any medication regularly?” with answers “Yes” and “No.” Participants’ functional health status is evaluated with the question “Have you ever had any problems with self-care-taking?” and the answers were “Yes” and “No.”

The ability of participants to perform daily actions, including taking a shower, moving about without physical limitations, performing home chores, using public transportation, and engaging in other activities like shopping, were the key indicators of self-care taking. To find out whether these risky behaviors are associated with the use of health services.

Statistical analysis

After data were extracted, it was revised, coded, and fed to statistical software using IBM SPSS version 22 (SPSS, Inc. Chicago, IL). All statistical analysis was done using two-tailed tests, and a P-value less than 0.05 was considered statistically significant. Descriptive analysis based on the frequency and percent distribution was done for all variables, including participants’ bio-demographic data, personal habits, perceived health status, and health-seeking behaviors. Univariant relations between participants' data and their perceived health status with health-seeking behaviors were shown using the crosstabulation method. The significance of association was tested using Pearson chi-square and exact probability tests in case of small frequency distributions.

## Results

A total sample of 481 participants completed the study questionnaire. Participants' ages ranged from 18 to above 60 years, with a mean age of 24.8. Two hundred fifty-six (53.2%) participants were females. Two hundred twenty-nine (47.6%) were single, and 241 (50.1%) were married. A monthly income of less than 4,000 Saudi riyals was reported among 223 (46.4%) participants, while 208 (43.2%) had a monthly income of 4,000-14,000 Saudi riyals. Exact 355 (73.8%) rate their economic status as intermediate and 34 (7.1%) as high. A total of 296 (61.5%) participants were not working, and only 65 (13.5%) were smokers, while four (0.8%) reported having alcohol (Table 1).

**Table 1:** Socio-demographic variables and behaviors of study participants, Al-Ahsa, Saudi Arabia

Socio-demographic variables	Number	Percentage
Age in years		
< 25	255	53.0%
25-35	112	23.3%
35-45	56	11.6%
45-60	50	10.4%
> 60	8	1.7%
Gender		
Male	225	46.8%
Female	256	53.2%
Marital status		
Single	229	47.6%
Married	241	50.1%
Divorced / widow	11	2.3%
Number of children		
None	47	18.7%
1-2	91	36.1%
3-4	58	23.0%
5+	56	22.2%
Educational level		
Below secondary	38	7.9%
Secondary	135	28.1%
University / above	308	64.0%
Residence		
Urban	222	46.2%
Rural	259	53.8%
Monthly income		
< 4000 Saudi riyals	223	46.4%
4000-14000 Saudi riyals	208	43.2%
> 14000 Saudi riyals	50	10.4%
How do you rate your economic situation?		
Low	92	19.1%
Intermediate	355	73.8%
High	34	7.1%
Work		
Not working	296	61.5%
Governmental sector	80	16.6%
Private sector	90	18.7%
Their own work	15	3.1%
Smoking		
Smoker	65	13.5%
Non-smoker	416	86.5%
Have alcohol		
Yes	4	.8%
No	477	99.2%

Exact 70.7% of females visit any healthcare center if they suffer from any health symptoms compared to 61.8% of males with recorded statistical significance (P=.038). Also, 73% of married participants do the visits compared to 54.5% of the widow/divorced group (P=.009). 68.5% of participants with intermediate economic status visit healthcare centers for any symptoms compared to 50% of others with high financial status (P=.049) (Table 2).

**Table 2:** Health-seeking behaviour by participants' socio-demographic variables

Socio-demographic variables	Do you visit any healthcare center if you suffer from any health symptoms?				P-value
	Yes		No		
	Number	Percentage (%)	Number	Percentage (%)	
Age in years					.247^$^
< 25	162	63.5%	93	36.5%	
25-35	74	66.1%	38	33.9%	
35-45	38	67.9%	18	32.1%	
45-60	40	80.0%	10	20.0%	
> 60	6	75.0%	2	25.0%	
Gender					.038*
Male	139	61.8%	86	38.2%	
Female	181	70.7%	75	29.3%	
Marital status					.009*
Single	138	60.3%	91	39.7%	
Married	176	73.0%	65	27.0%	
Divorced / widow	6	54.5%	5	45.5%	
Educational level					.309
Below secondary	21	55.3%	17	44.7%	
Secondary	91	67.4%	44	32.6%	
University / above	208	67.5%	100	32.5%	
Residence					.655
Urban	150	67.6%	72	32.4%	
Rural	170	65.6%	89	34.4%	
Monthly income					.245
< 4000 Saudi riyals	152	68.2%	71	31.8%	
4000-14000 Saudi riyals	140	67.3%	68	32.7%	
> 14000 Saudi riyals	28	56.0%	22	44.0%	
How do you rate your economic situation?					.049*
Low	60	65.2%	32	34.8%	
Intermediate	243	68.5%	112	31.5%	
High	17	50.0%	17	50.0%	
Work					.340
Not working	199	67.2%	97	32.8%	
Governmental sector	58	72.5%	22	27.5%	
Private sector	54	60.0%	36	40.0%	
Their own work	9	60.0%	6	40.0%	

73.3% of participants diagnosed with a disease or chronic diseases visited a healthcare center for clinical symptoms compared to 64.3% of others without (P=.049). Also, 78.2% of those who had any trouble with their daily activities visited healthcare centers compared to 64.3% of others who had no problems (P=.017). 69% of non-smokers visited healthcare centers versus 50.8% of smokers (P=.004) (Table 3).

**Table 3:** Distribution of participants health seeking behavior by their perceived health status

Perceived health status	Do you visit any healthcare center if you suffer from any health symptoms?				P-value
	Yes		No		
	Number	Percentage (%)	Number	Percentage (%)	
How do you rate your general health?					.736^$^
Bad	3	60.0%	2	40.0%	
Intermediate	13	65.0%	7	35.0%	
Good	47	73.4%	17	26.6%	
Very good	145	66.8%	72	33.2%	
Excellent	112	64.0%	63	36.0%	
Have you ever been diagnosed with a disease or chronic disease?					.049*
Yes	88	73.3%	32	26.7%	
No	232	64.3%	129	35.7%	
Has anyone in your family been diagnosed with a chronic disease or disease?					.170
Yes	215	68.7%	98	31.3%	
No	105	62.5%	63	37.5%	
Do you take any medications regularly?					.181
Yes	79	71.8%	31	28.2%	
No	241	65.0%	130	35.0%	
Have you had any trouble with your daily activities?					.017*
Yes	61	78.2%	17	21.8%	
No	259	64.3%	144	35.7%	
Smoking					.004*
Smoker	33	50.8%	32	49.2%	
Non-smoker	287	69.0%	129	31.0%	

## Discussion

To the best of our knowledge, this is the first study to understand healthcare-seeking behaviors and related factors in Al-Ahsa, Saudi Arabia. In evaluating the factors influencing health-seeking behavior, Income level and general health (including previously diagnosed chronic diseases and smoking habits) were significant in our study. In the present study, almost half of the participants were under 25. This contradicts other studies, which showed that men aged 60-69 years and women aged 70 years and over were more likely to report healthcare-seeking than younger responders [12]. This may be due to electronic surveys in our study, and younger participants will more easily fulfill electronic data collection and questionnaires. In addition, we found that half of the participants were women. This concurs with another study done in Canada that examined the means and standard deviations that indicated that women reported they would visit their primary care physician in response to health concerns to a greater extent than men [13].

In this study, the majority who are diagnosed with chronic diseases seek medical care centers for any symptom, consistent with the study by Dewa Adhikari and Dagendra Prasad Rijal that found that more than half of the major chronic patients seek medical care [14]. Similarly, another study done in Turkey agrees that chronic conditions substantially impact health-seeking behavior [11].

Socioeconomic factor plays an important role in the preference for health-seeking behavior. The present study found that the relationship between patients with intermediate economic status and the increasing number of visits to the primary healthcare center for any symptoms is statistically significant compared to the high-income patient. Medical Insurance has a crucial role in this aspect. High-income patients are more likely to be medically covered than middle income. As a result, a medically covered patient is more likely to visit a private healthcare center. This result is consentient with a study done in South Africa which found that patients with medical insurance are more likely to seek private healthcare centers when becoming ill [15].

In contrast, a study conducted in Saudi Arabia found that monthly income is not related to healthcare utilization [16]. The disparity between the results may be attributed to differences in educational level and demographic factors between the regions across Saudi. Another study conducted in Belgium found that the higher usage of healthcare centers among lower-income patients is due to differences in health needs. This is explained by the presence of vertical equity in the usage of primary healthcare services among this group. This means the greater the needs of the service are met by greater use [17].

Half of the respondents preferred public healthcare centers over private healthcare centers. This result is consistent with a study that measured patient trust in doctors, which found that Saudi Arabian patients have a higher confidence level toward doctors working in public hospitals than in private hospitals [18]. However, another study conducted in the eastern region of Saudi found that private hospitals provide better quality than public care services. This is due to the high concentration of private facilities promoting their service for profit compared to public ones, which have a non-profit approach [19].

Nearly one-fourth of participants went to the health center for exams even though they had no symptoms, and also, nearly a quarter chose not to go even though they knew they required medical care. We assume that the difference in the ratio is due to self-treatment practice and sociodemographic factors. People who live in central cities are more accessible to healthcare centers and consequently more able to do regular medical checkups. In contrast, people who live in rural areas depend more on pharmaceutical/ herbal treatment. This result is consistent with a study done in Vietnam, which shows that limited access to healthcare in mountainous areas has a rule in decreasing medical checkups and increasing dependency on self-treatment practice [20].

## Conclusions

This information is important to help the government and policymakers create an effective strategy and manage the existing resources in Al-Ahsa. We found that most participants diagnosed with chronic diseases seek medical care for any symptom. Also, the intermediate economic status patients are more going to the government primary healthcare center for any symptoms. These findings highlight the need for more national research and give evidence for particular initiatives to eliminate socioeconomic disparities in healthcare utilization.
